# Structure and function of urea amidolyase

**DOI:** 10.1042/BSR20171617

**Published:** 2018-01-17

**Authors:** Jing Zhao, Li Zhu, Chen Fan, Yi Wu, Song Xiang

**Affiliations:** 1 Key Laboratory of Nutrition and Metabolism, Institute for Nutritional Sciences, Shanghai Institutes for Biological Sciences, Chinese Academy of Sciences, Shanghai 200031, China; 2 School of Life Sciences, Lanzhou University, Lanzhou 730000, China

**Keywords:** biotin, crystallography, hydrolases, nitrogen metabolism, urea amidolyase

## Abstract

Urea is the degradation product of a wide range of nitrogen containing bio-molecules. Urea amidolyase (UA) catalyzes the conversion of urea to ammonium, the essential first step in utilizing urea as a nitrogen source. It is widely distributed in fungi, bacteria and other microorganisms, and plays an important role in nitrogen recycling in the biosphere. UA is composed of urea carboxylase (UC) and allophanate hydrolase (AH) domains, which catalyze sequential reactions. In some organisms UC and AH are encoded by separated genes. We present here structure of the *Kluyveromyces lactis UA* (KlUA). The structure revealed that KlUA forms a compact homo-dimer with a molecular weight of 400 kDa. Structure inspired biochemical experiments revealed the mechanism of its reaction intermediate translocation, and that the KlUA holo-enzyme formation is essential for its optimal activity. Interestingly, previous studies and ours suggest that UC and AH encoded by separated genes probably do not form a KlUA-like complex, consequently they might not catalyze the urea to ammonium conversion as efficiently.

## Introduction

Degradation of a wide range of nitrogen-containing bio-molecules generates urea. Mammals do not directly utilize urea, and excrete it into the environment. In contrast, many plants, fungi and bacteria can utilize urea as a nitrogen source [1–3]. They reintegrate its nitrogen into the biosphere, playing important roles in the nitrogen recycling within the biosphere. The first step of their urea utilization is the conversion of urea to ammonium, which is catalyzed by two distinct enzymes, urease and urea amidolyase (UA) [4]. UA is widely distributed in fungi, bacteria and other microorganisms [5,6]. In many UA containing organisms the Ni^2+^-dependent urease is not found, neither is the Ni/Co co-transporter. These organisms might have dropped all Ni-dependent pathways during evolution, and have gained a selective advantage since Ni^2+^ can be toxic if its cellular level is not tightly regulated [5]. In addition to urea utilization, UA also plays important roles in a recently discovered eukaryotic pyrimidine nucleic acid precursor degradation pathway [7], and the pathogenesis of human pathogens such as *Candida albicans* [8–10].

**Figure 1:**
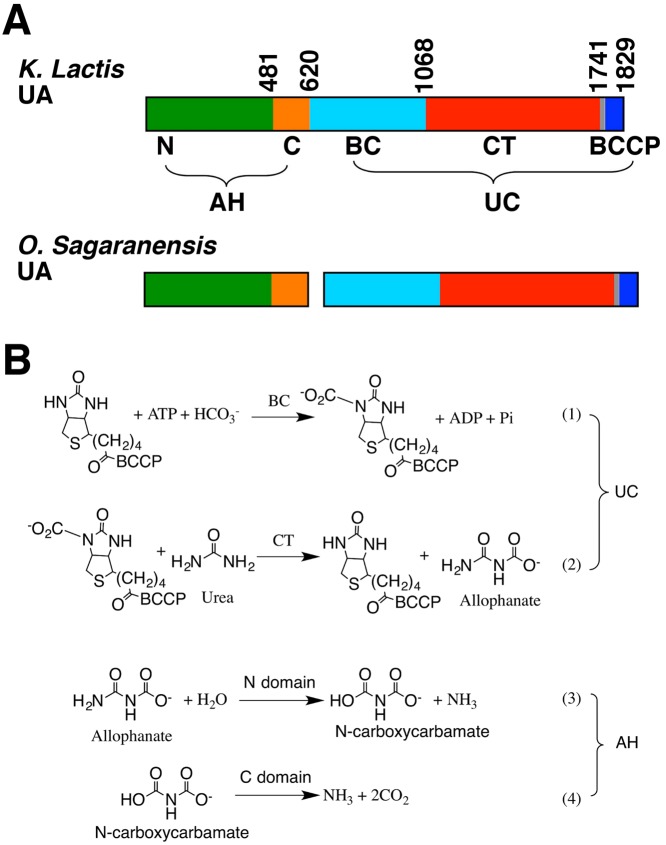
Domain architecture and reactions catalyzed by UA. (**A**) Domain architecture of UA and the related UC and AH proteins. Domains are colored coded. The color scheme is used throughout the paper unless otherwise indicated. Domain boundaries for KlUA are shown. (**B**) Reactions catalyzed by UA.

UA is composed of urea carboxylase (UC) and allophanate hydrolase (AH) domains. UC converts urea to allophanate, and AH subsequently converts it to ammonium [11]. UC belongs to the biotin-dependent carboxylase family. Biotin is covalently linked to its biotin-carboxyl carrier protein (BCCP) domain. Its biotin carboxylase (BC) and carboxyltransferase (CT) domains catalyze sequential reactions in urea caboxylation [12,13]. The AH domain is composed of N and C domains, which catalyze sequential reactions in the allophanate to ammonium conversion [14] (Figure 1A,B). In some organisms, UC and AH are encoded by separated genes [5,6,15,16]. Phylogenetic analyses suggest that the UA gene is the result of a fusion event of AH and UC genes [6].

Structural characterizations of UC [17] and AH [14,18,19] have provided insights into their catalytic mechanism. However, the architecture of the UA holo-enzyme is not clear, how UC and AH coordinate in the holo-enzyme is poorly understood. We present here crystal structure of *Kluyveromyces lactis* UA (KlUA) at 6.5 Å resolution. The structure revealed that KlUA forms a compact dimer with a molecular weight of 400 kDa. Structure guided biochemical experiments provided insights into allophanate translocation between the UC and AH domains, and indicated that the KlUA holo-enzyme formation is required for its optimal activity. Previous studies and ours suggest that UC and AH encoded by separated genes probably do not form a KlUA-like complex. Consequently, they probably catalyze the urea to ammonium conversion less efficiently.

## Experimental procedures

### Protein expression and purification

The KlUA gene was amplified from the *Kluyveromyces lactis* genome, and inserted into vector pET28A (Novagen). To generate the KlUAΔBCCP construct, a stop codon was inserted after base pair 5223. This plasmid was transformed into *Escherichia coli* BL21 (DE3) Star cells, which were cultured in LB medium supplemented with 50 mg/l kanamycin, and induced with 0.3 mM isopropyl β-D-1-thiogalactopyranoside when the optical density at 600 nm reaches 1.0, at 16°C for 16 hours. The KlUAΔBCCP protein was purified by nickel-nitrilotriacetic acid (Qiagen), anion-exchange (Hitrap Q HP, GE Healthcare) and size exclusion columns (Sephacryl S300 HR, GE Healthcare). Purified protein was concentrated to 10 mg/ml, in a buffer containing 20 mM Tris/HCl (pH 8.0), 200 mM NaCl, 2 mM DTT and 5% glycerol, flash cooled in liquid nitrogen and stored at −80°C.

The expression and purification of the full length KlUA, *Kluyveromyces lactis* UC (KlUC) and *Kluyveromyces lactis* AH (KlAH) followed protocols reported previously [14,17].

The KlUA mutants were generated with the QuikChange kit (Agilent Technologies) and verified by DNA sequencing. The mutants were expressed and purified following the same protocol for the wild type protein.

### Crystallization and structure determination

Rod-shaped crystals were obtained with the sitting drop vapor diffusion method. The crystallization experiments were conducted at 4°C, and the reservoir solution contains 0.1 M Tris/HCl (pH 7.5), 0.2 M ammonium sulfate, 12% PEG 8000, 2% PEG3350. Before crystallization experiments the protein solution was supplemented with 0.5 mM urea, 0.5 mM ADP and 0.5 mM sodium malonate (pH 7.0). For data collection the crystals were equilibrated in the reservoir solution supplemented with 20% glycerol, flash cooled and stored in liquid nitrogen.

Diffraction data were collected at 100 K, on an ADSC Q315 charge-coupled device detector at the Shanghai Synchrotron Radiation Facility beamline BL17U, at a wavelength of 1.0391 Å (Table 1). The data were processed with mosflm [20] and scaled with scala [21]. The structure of KlUAΔBCCP was determined with molecular replacement with phaser [22], using the structures of KlUC (PDB 3VA7) and KlAH (PDB 4ISS) as search models. Inspection and manipulation of the structure were carried out with coot [23] and O [24]. Structural refinement was carried out with phenix [25]. Mosflm, scala and phaser are programs in the CCP4 suite [26].

**Table 1:** Data collection and refinement statistics

**Data collection**	
Space group	P2_1_2_1_2_1_
Cell dimensions	
*a, b, c* (Å)	105.7, 181.9, 549.8
*α, β, γ* (°)	90.0, 90.0, 90.0
Wavelength	1.0391
Resolution (Å)	30.0–6.5 (6.85–6.5)
*R*_merge_	0.118 (0.581)
*I*/σ*I*	8.3 (2.2)
Completeness (%)	95.6 (95.6)
Redundancy	3.9 (4.0)
**Refinement**	
Resolution (Å)	30.0–6.5 (6.84–6.5)
No. reflections	20603 (2740)
*R*_work/_ *R*_free_	27.8/30.2 (34.4/38.1)
No. atoms	51722
Average B-factor (Å^2^)	308.4
R.m.s. deviations	
Bond lengths (Å)	0.009
Bond angles (°)	1.638

*Numbers in parentheses are for the highest resolution shell.

### Negatively stained electron microscopy

Full-length KIUA was diluted to 1 mg/ml in a buffer containing 20 mM Tris (pH 7.5), 110 mM NaCl, 0.2 mM DTT, 5 mM ADP and 5 mM urea. Following 15 minutes of incubation on ice, the samples were further diluted, applied onto glow-discharged, thin carbon-film covered copper grid (300-mesh) as 3 μl droplets. The grids were blotted, stained with 1% uranyl acetate for 30 seconds and observed with a FEI G2 electron microscope operating at 200 kV. Micrographs were recorded by SO 163 films at a nominal magnification of 50000×, and scanned at 2000 dpi with a pixel size of 2.54 Å. 4495 particles were manually selected using e2boxer.py [27]. Alignment and 2D classification were performed by RELION [28].

### Enzyme kinetic assays

The UA activity was assayed by monitoring the production of ammonium, which is coupled to the NADH to NAD conversion by glutamate dehydrogenase [29]. The reaction mixture contained 100 mM Tris/HCl (pH 8.0), 1 μM EGTA, 20 mM potassium chloride, 11 mM magnesium choloride, 5 mM ATP, 12.5 mM sodium bicarbonate, 50 mM oxoglutarate, 0.3 mM NADH, 100 units/ml glutamate dehydrogenase, 0.15 μM KlUA or related proteins and variable concentrations of urea. For reactions containing two proteins, 0.15 μM of each is used unless otherwise indicated.

The AH activity was assayed similarly. The reaction mixture contained 100 mM Tris/HCl (pH8 .0), 1 μM EGTA, 20 mM potassium choloride, 6 mM magnesium choloride, 50 mM oxoglutarate, 0.3 mM NADH, 100 units/ml glutamate dehydrogenase, 0.05 μM KlUA or related proteins, and variable concentrations of potassium allophanate. Potassium allophanate was produced as described [16].

### Analytical gel filtration

Analytical gel filtration experiments were performed on a superpose 6 HR column (GE Healthcare). 0.25 mg of protein samples was injected on the column, and eluted with a buffer containing 20 mM Tris/HCl (pH 8.0) and 200 mM NaCl. The column was calibrated with protein standards (Sigma–Aldrich) including carbonic anhydrase (29 kDa), bovine serum albumin (66 kDa), alcohol dehydrogenase (150 kDa), β-amylase (200 kDa), apoferritin (443 kDa) and bovine thyroglobulin (669 kDa).

## Results

### Structure determination

The full-length KlUA failed to crystallize. We speculated that the mobility of the BCCP domain prevented its crystallization, and hence removed it. This variant (residues 1–1741, KlUAΔBCCP) crystallized with the vapor diffusion method. Despite extensive efforts, the best crystals only diffract to 6–7 Å resolution at the Shanghai Synchrotron Radiation Facility. They belong to space group P2_1_2_1_2_1_ and contain four copies of KlUAΔBCCP in the asymmetric unit. The structure was determined with molecular replacement, using structures of KlUC [17] and KlAH [14] as search models. The 100% sequence identity between the search models and the crystalized protein facilitated subsequent refinements. The structure was refined to a resolution of 6.5 Å, and agrees well with the crystallographic data, and expected geometric values (Table 1). At this resolution the structure did not reveal any significant differences between the structures of its UC and AH domains and the previously reported KlUC and KlAH structures.

**Figure 2:**
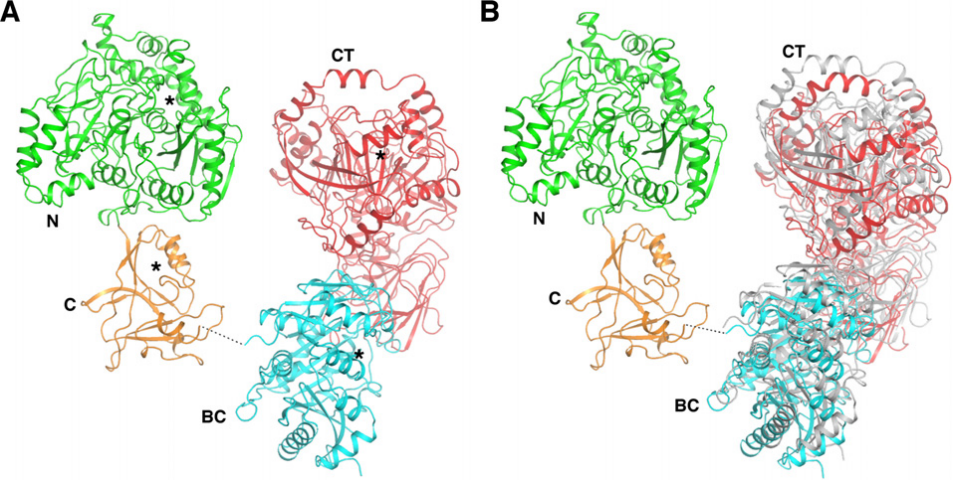
Structure of KlUA. (**A**) Structure of the KlUA monomer. The black stars indicate active sites. (**B**) Structural comparison of monomers in the crystal. The AH domains are aligned, and the UC domains with the most different orientations are shown, one is color coded as in Figure 1A and the other is colored in gray. Structural figures were generated with pymol (http://www.pymol.org).

The linker between the AH and UC domains is not clearly defined in the electron density map. However, in the crystal near each AH C-terminus only one UC N-terminus is found, indicating that they belong to the same polypeptide (Figure 2A). The relative orientations of the AH and UC domains in the four KlUAΔBCCP protomers are very similar. With the AH domains aligned, the positions of the UC domains are related by rotations of 10° or less (Figure 2B). There are few contacts between the UC and AH domains in the same polypeptide.

### Architecture of the KlUA holo-enzyme

**Figure 3:**
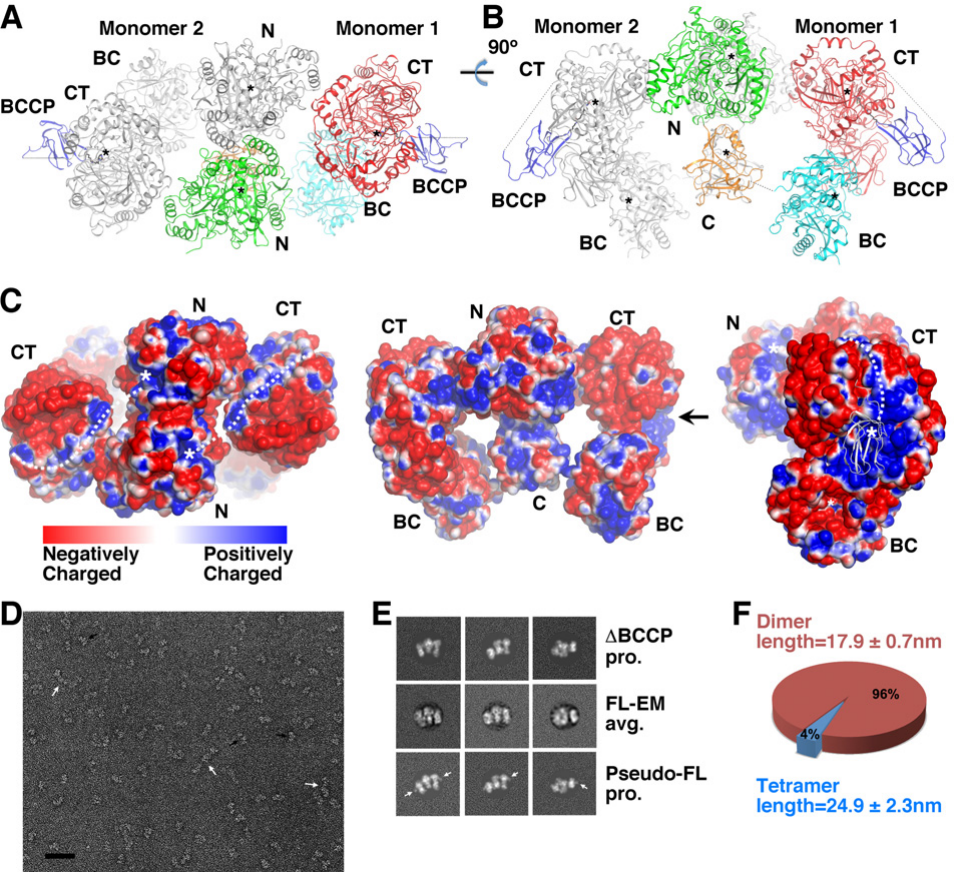
Architecture of the KlUA complex. (**A**) and (**B**) Architecture of the dimeric KlUA complex. The black stars indicate the active sites. The BCCP domains is modeled and shown in blue. In panel (**A**) one looks down the pseudo two-fold axis. The structure presented in panel (**B**) resembles the letter ‘m’. (**C**) Surface charge distribution of KlUA. The orientations of the left and middle panels are identical with that in Figure 3A,B. In the right panel one views along the arrow indicated in the middle panel. In this panel the modeled BCCP domain is shown in cartoon representation and colored in white. The white stars indicate the active sites. The dashed white lines in the left and right panels indicate the positively charged surface patch connecting the active sites of the UC CT and AH N domains. (**D**) Representative negative stained electron microscopy micrograph of KlUA. Dimers and tetramers are indicated by black and white arrows, respectively. The scale bar represents 50 nm. (**E**) Representative class average of full-length KIUA (FL-EM avg.). Projections of the KIUAΔBCCP crystal structure (ΔBCCP proj.) and a full-length KIUA structure with modeled BCCP domains (Pseudo-FL proj., the modeled BCCP domains are indicated by white arrows) are shown for comparison. (**F**) Size distribution of the observed particles. The ratio of dimer and tetramer is calculated from a total of 613 particles in eight micrographs.

In the crystal the four KlUAΔBCCP polypeptides are organized into two very similar dimers. The structure of the dimer resembles the letter ‘m’, with the AH domains at the center, sandwiched by the UC domains on both sides. The two monomers are roughly related by a two-fold rotational symmetry (Figure 3A–C). Although the BCCP domain is not present in the protein we crystallized, the structure of KlUC [17] allowed us to model it onto the observed KlUAΔBCCP dimer. This indicates that the BCCP domains are located at opposite ends of the KlUA dimer (Figure 3A,B), which would allow it to move freely to deliver carboxyl-biotin between the BC and CT domains, consistent with its role in the catalysis.

To confirm the physiological relevance of the observed dimer in the crystal, we characterized the architecture of full-length KlUA by negatively stained electron microscopy (Figure 3D). Images of mono-dispersed KIUA particles were subjected to alignment and 2D classification. The dimension and appearance of this average corresponds well to the distinctive, ‘m’-shaped projection of the dimeric KIUAΔBCCP crystal structure. However no density in the class averages could be reliably assigned to domain BCCP, presumably due to its conformational flexibility (Figure 3E). A small number of larger particles were also observed, and their sizes suggest that they correspond to a tetrameric form of KlUA. These particles showed prominent structural variability, suggesting that they result from weak and non-specific association of the KlUA dimers. The prevalence of the ‘m’-shaped particles observed in the electron microscopy visualization (Figure 3F) suggests that full-length KIUA in solution adopts a dimeric structure similar to that of KIUAΔBCCP in the crystal.

### Interactions between the KlUA monomers

**Figure 4:**
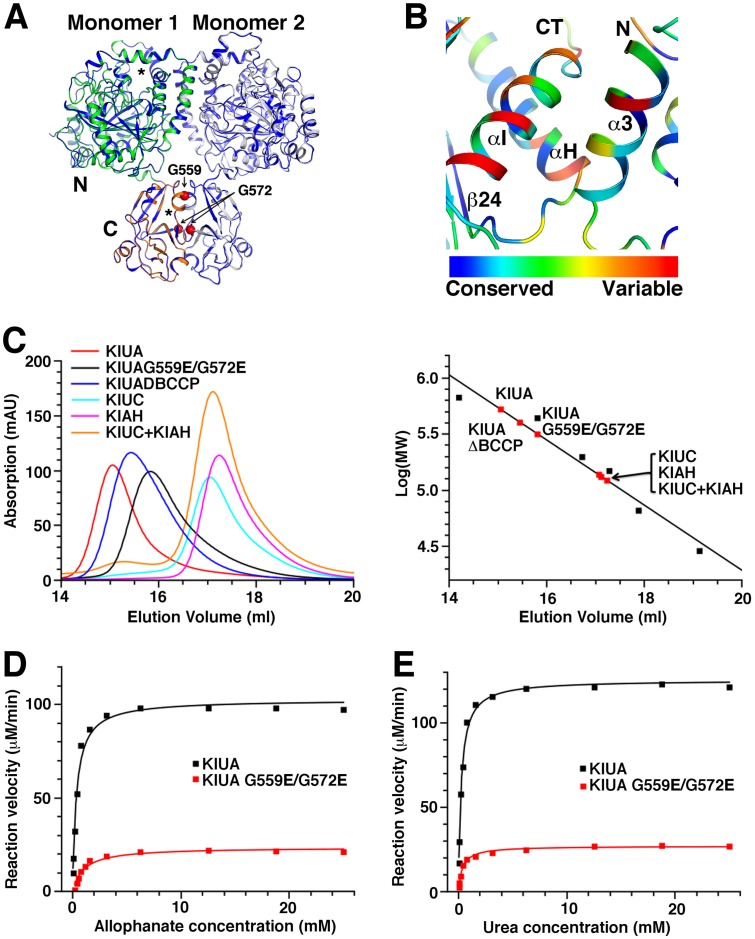
Interactions between the monomers in the KlUA holo-enzyme. (**A**) Interactions between the AH domains. One AH domain is color coded as in Figure 1A, the other is shown in gray. The structure of the isolated KlAH dimer (PDB 4ISS, blue) is shown for reference. The black stars indicate the active sites of the N and C domains. The red spheres indicate locations of Gly559 and Gly572. (**B**) Interactions between the UC CT and AH N domains. Secondary structure elements participating in the interaction are labeled. The structure is colored according to the conservation of individual residues among UA from different species. (**C**) Analytical gel filtration analysis of KlUA and related proteins. The column is calibrated with standards with molecular weights ranging from 29 kDa to 669 kDa (black squares in the right panel). (**D**) and (**E**) AH (**D**) and UA (**E**) activities of KlUA and its G559E/G572E mutant.

In the KlUA dimer, the interactions between the monomers are located at two interfaces. One is between the AH domains, which form a dimer identical with the isolated KlAH [14] (Figure 4A). This interface is composed of highly conserved residues and buries 5000 Å^2^ of surface area. The other is between the UC CT domain and the AH N domain from the other polypeptide. This interface is mainly contributed by helices αH and αI in the UC CT domain, and helix α3 in the AH N domain, burying 1000 Å^2^ of surface area. Residues at this interface are not very conserved among UA from different species [14,17] (Figure 4B).

To probe the physiological relevance of the interface between the AH domains, we introduced the G559E/G572E mutation that renders the isolated KlAH monomeric [14], and determined its effect on the KlUA holo-enzyme formation by analytical gel filtration experiments. In these experiments, the full-length KlUA has an apparent molecular weight of 500 kDa (the molecular weight of the monomeric KlUA is 202 kDa), and KlUAΔBCCP has an apparent molecular weight of 400 kDa (the molecular weight of the monomeric form is 190 kDa, Figure 4C), indicating that they both form homo-dimers. In contrast, the G559E/G572E mutant appeared to be monomeric, with an apparent molecular weight of 310 kDa. Therefore, the extensive interface between the AH domains is essential for the UA holo-enzyme assembly.

To assess the contribution of the UC–AH interface in the holo-enzyme formation, we tested whether isolated KlUC and KlAH form a stable complex in solution. KlUC and KlAH are monomeric and dimeric in solution, respectively [14,17]. They have similar molecular weights and elute at roughly the same volume in our analytical gel filtration experiments. Their mixture elutes at the same volume, indicating that they do not form a stable complex in solution (Figure 4C). The relatively smaller UC–AH interface therefore does not play a major role in the UA holo-enzyme assembly.

In the isolated KlAH, the active sites are located near the dimer interface. The extensive interactions at the dimer interface most likely stabilize the structure of the active sites. Consistent with this, the G559E/G572E mutation that renders the isolated KlAH monomeric severely inhibited its activity [14]. The AH domains in KlUA form an identical dimer, and the G559E/G572E mutation that renders the enzyme monomeric also severely reduces its AH activity (Figure 4D and Table 2) and its overall activity (Figure 4E and Table 2). The interactions between the AH domains in the KlUA holo-enzyme probably also stabilize the active site structures of the AH domain, and is required for its optimal activity.

**Table 2:** Summary of kinetic parameters

	*k*_cat_ (min^−1^)	*K*_m_^1^ (mM)	*k*_cat_*/K*_m_ (min^−1^ mM^−1^)
UA activity			
KlUA	836 ± 11 (1.0)^2^,^3^	0.255 ± 0.018 (1.0)	3278 ± 276 (1.0)
KlUA (S177A)	47.1 ± 0.8 (0.056)	0.203 ± 0.020 (0.80)	232 ± 27 (0.071)
KlUA (K1605A)	79.7 ± 2.0 (0.095)	0.299 ± 0.039 (1.17)	267 ± 41 (0.081)
KlUA (G559E/G572E)	180.2 ± 3.2 (0.22)	0.361 ± 0.032 (1.42)	499 ± 54 (0.15)
KlUA (S177A) + KlUA (K1605A)	761 ± 19 (0.91)	0.216 ± 0.029 (0.85)	3524 ± 562 (1.08)
KlUC+KlAH	604 ± 7.9 (0.72)	0.246 ± 0.017(0.96)	2456 ± 205 (0.75)
KlUA (S177A) + KlAH	602 ± 10 (0.72)	0.363 ± 0.030 (1.43)	1655 ± 164 (0.51)
KlUA (K1605A) + KlUC	786 ± 11 (0.94)	0.254 ± 0.019 (1.0)	3096 ± 268 (0.94)
KlUA + 5x KlUC	1368 ± 43 (1.64)	0.188 ± 0.033 (0.74)	7898 ± 1509 (2.22)
KlUA + 5x KlAH	1184 ± 22 (1.42)	0.300 ± 0.029 (1.18)	3948 ± 457 (1.20)
AH activity			
KlUA	2050±41 (1.0)	0.36±0.037 (1.0)	5695±700 (1.0)
KlUA (G559E/G572E)	471±22 (0.23)	1.04±0.183 (2.88)	453±101 (0.080)
KlAH	1523±43 (0.74)	0.432±0.060 (1.2)	3527±592 (0.62)

1 The *K*_m_ values are for urea (UA activity) and allophanate (AH activity).

2 Errors were obtained from fitting the experimental data to the Michaelis–Menten equation.

3 Numbers in parentheses are ratios to the wild type values.

### Translocation of allophanate

During the UA catalysis, allophanate is produced at the active site of the UC CT domain and is translocated to that of the AH N domain for subsequent reaction. In the KlUAΔBCCP structure, these active sites are located more than 70 Å apart. A somewhat continuous positively charged surface patch connects these active sites in the same polypeptide (Figure 3C). Allophanate is negatively charged at physiological pH, and this prompted us to test if this patch mediates substrate channeling of allophanate within the same KlUA polypeptide, which can greatly reduce the transit time of reaction intermediates [30,31].

**Figure 5:**
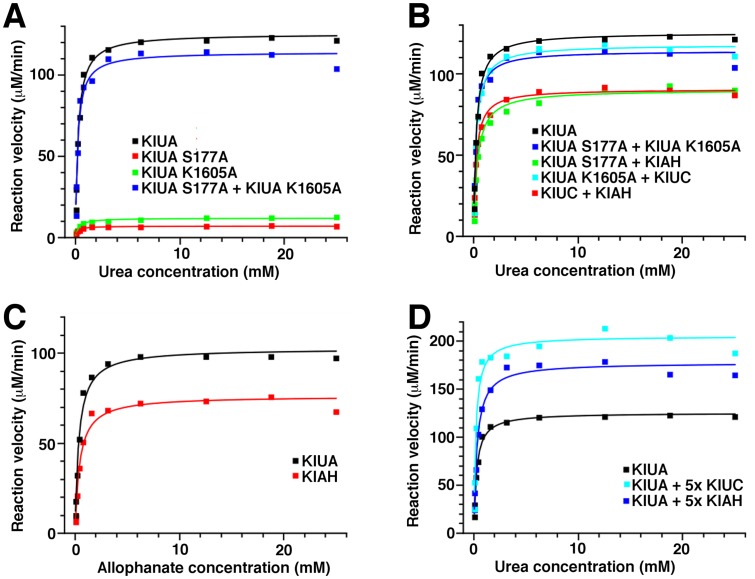
Kinetic assays of KlUA and related proteins. (**A**), (**B**) and (**D**) The UA activity of KlUA and related proteins/mixtures. (**C**) The AH activity of KlAH and full-length KlUA.

If allophanate is channeled between active sites in the same KlUA polypeptide, only a KlUA polypeptide with intact UC and AH active sites can catalyze the urea to ammonium conversion. Otherwise, a UA polypeptide with defective UC activity can cooperate with one with defective AH activity for the catalysis. To test if substrate channeling within the same KlUA polypeptide takes place, we mixed the K1605A and S177A mutants of KlUA at 1:1 molar ratio and tested if the mixture can catalyzes urea to ammonium conversion. The conserved Lys1605 side chain in the UC CT active site most likely serves as the essential general base for the CT reaction, and the K1605A mutation severely inhibited the activity of the isolated KlUC [17]. The conserved Ser177 side chain hydroxyl in the AH N domain active site performs the initial nucleophilic attack on allophanate, and the S177A mutation inactivates the isolated KlAH [14]. These mutations should inhibit the UC and AH activities of UA, respectively. Consistently, neither mutant was able to catalyze the urea to ammonium conversion efficiently (Figure 5A). In contrast, their mixture was able to catalyze the urea to ammonium conversion as efficiently as the wild type KlUA (Figure 5A and Table 2). Therefore at least in our experiment setup, substrate channeling of allophanate within the same KlUA polypeptide does not play a major role.

KlUA is dimeric in solution and substrate channeling of allophanate could take place between different polypeptides within the holo-enzyme. If the dissociation and re-association of the KlUA dimer is rapid, mixing the K1605A and S177A mutants at 1:1 molar ratio will generate a K1605A/S177A hybrid dimer, constituting 50% of the KlUA dimer population. If allophanate is channeled between different polypeptides in the KlUA dimer, this is the only active dimer species, and it has to catalyze the urea to ammonium conversion twice as fast as the wild type KlUA to produce the effect we saw in Figure 5A. Additional experiments (see discussion) indicate that it is not likely to be the case. Rather, Figure 5A is consistent with a model that allophanate is translocated from the active site of UC CT domain to that of the AH N domain via diffusion through solvent.

### The UA holo-enzyme catalyzes the urea to ammonium conversion more efficiently

The KlUA holo-enzyme forms a tight complex. Although substrate channeling of allophanate is unlikely to take place within the KlUA holo-enzyme, we found that it catalyzes the urea to ammonium conversion more efficiently. Compared with the mixture of isolated KlUC and KlAH, its catalysis is approximately 30% faster (Figure 5B and Table 2).

We tested the possibility that this is due to enhanced AH or UC activities of the KlUA holo-enzyme. To compare the activity of the AH domain in KlUA and the isolated KlAH, we compared the urea to ammonium conversion activity of two mixtures: one contains KlUA mutants S177A and K1605A, the other contains KlUA mutant S177A and isolated KlAH. In both mixtures the same protein catalyzes the UC reaction (KlUA mutant S177A). In the first mixture the AH reaction is catalyzed by the AH domain in the KlUA K1605A holo-enzyme, whereas in the second it is catalyzed by the isolated KlAH. The reaction catalyzed by the first mixture was approximately 30% faster, indicating that the AH domain in the KlUA K1605A holo-enzyme is more active than the isolated KlAH (Figure 5B and Table 2). Measuring the AH activity of KlUA and isolated KlAH directly also indicates that the AH activity of KlUA is about 30% stronger (Figure 5C and Table 2). Therefore the KlUA holo-enzyme enhances its AH activity.

We performed a similar experiment to compare the activities of isolated KlUC and the UC domain in the KlUA holo-enzyme. This experiment revealed that the urea to ammonium conversion activities of the KlUC/KlUA K1605A and KlUA S177A/KlUA K1605A mixtures were roughly the same (Figure 5B and Table 2), suggesting that either the isolated KlUC has the same activity as the UC domain in the KlUA S177A holo-enzyme, or the AH reaction is rate-limiting. To test if the AH or the UC reaction is rate-limiting, we artificially increased the UC and AH activities by supplementing KlUC and KlAH, respectively. If one of these reactions is rate-limiting, increasing its activity should cause an increase in the overall UA reaction rate, whereas increasing the activity of the other should not have an effect. The experiments revealed that increasing either the UC or the AH activities caused significant increases in the overall UA reaction rate (Figure 5D and Table 2), indicating that no rate-limiting step in the KlUA catalysis could be detected under the experimental conditions we used. Therefore, the activity of the isolated KlUC is not significantly different from that of the UC domain in the KlUA S177A holo-enzyme. Assuming that the S177A mutation at the AH N domain active site does not affect the UC activity, these data indicate that the KlUA holo-enzyme formation does not affect its UC activity.

## Discussion

Our crystallographic and electron microscopic studies on KlUA provided a clear picture of its holo-enzyme architecture. The formation of the dimeric KlUA holo-enzyme is mediated by extensive and conserved interactions between its AH domains, and relatively weaker, less conserved interactions between its UC and AH domains. If there is no covalent linkage between the AH and UC domains, the latter is not strong enough to hold them to form a stable complex. This suggests that AH and UC encoded by separated genes probably do not form a KlUA-like complex, consistent with studies on a number of such AH and UC proteins [16,18,32].

Although a continuous positively charged surface patch connects the active sites of the UC CT domain and the AH N domain in the same KlUA polypeptide, our data argue against a model that it mediates substrate channeling of allophanate. Instead, our data suggest that allophanate translocation is most likely via diffusion through solvent. The KlUA K1605A/KlUC mixture and the KlUA S177A/KlAH mixture can both catalyze the urea to ammonium conversion efficiently (Figure 5B). In these mixtures the KlUA holo-enzyme lacks either the UC or the AH activity. Therefore translocation of allophanate within the KlUA holo-enzyme cannot sustain the UA catalysis, and the observed the UA activity is entirely contributed by allophanate translocation between KlUA and isolated KlUC/KlAH, via diffusion through solvent. In addition, supplementing KlUA with isolated KlUC or KlAH can significantly increase the overall urea to ammonium conversion rate (Figure 5D), indicating that KlUA can take allophanate from solution (produced by the isolated KlUC) and release it to solution (to the isolated KlAH). Therefore, substrate channeling of allophanate within the same KlUA polypeptide or KlUA holo-enzyme is unlikely to play a role in the catalysis. A recent study on the *Pseudomonas syringae* UC and AH also ruled out the possibility that they form a transit complex to facilitate substrate channeling of allophanate during the catalysis [32].

KlUA forms a tight complex. Our study indicates that it catalyzes the urea to ammonium conversion more efficiently, compared with the mixture of isolated KlUC and KlAH. Subsequent analysis indicates that this is due to enhanced AH activity in the KlUA holo-enzyme, whereas the UC activity may not be affected by the holo-enzyme formation. The enhancement of the AH activity might be partly contributed by the interactions between the AH and UC domains, which could stabilize the structure of the AH domain. In addition, the positively charged surface patch on the UC domain near the AH N domain active site (Figure 3C) might facilitate the recruitment of allophanate, and enhance the AH activity. This suggests that the single-polypeptide form of UA is a more efficient nano-machine, compared with those composed of separated UC and AH proteins. The UA gene is most likely the result of a fusion event of the AH and UC genes. Our data indicate that such fusion produces a more efficient UA, therefore might confer a selective advantage in evolution. Gene fusion is among the most commonly utilized means by nature to generate novel genes, and it has been suggested that fusion events do not occur randomly and confer selective advantage [33,34]. Our study on KlUA provides an interesting example in this regard.

The AH domain plays a central role in the KlUA holo-enzyme formation. It is located at the center of the holo-enzyme and mediates most of the interactions between KlUA polypeptides. Its activity is enhanced by interactions with other domains in the holo-enzyme, which probably stabilizes its structure and/or facilitates its substrate recruitment. In many microorganisms, AH (also called AtzF and TrzF) is involved in the cyanuric acid degradation pathway [35–37], and forms a complex with AtzD (cyanuric acid amidohydrolase) and AtzE (biuret amidohydrolase), enzymes upstream in this pathway [19]. It would be interesting to see what role it plays in the formation of this complex, and if its activity is enhanced by this complex.

## Accession Numbers

The structure factors and coordinates for the KlUAΔBCCP structure have been deposited into the Protein Data Bank, with accession code 5I8I.

## Abbreviations

AH: allophanate hydrolase
BC: biotin carboxylase
BCCP: biotin-carboxyl carrier protein
CT: carboxyltransferase
KlAH: *K. lactis* AH
KlUA: *K. lactis* UA
KlUC: *K. lactis* UC
UA: urea amidolyase
UC: urea carboxylase
